# Confinement Correction to Mercury Intrusion Capillary Pressure of Shale Nanopores

**DOI:** 10.1038/srep20160

**Published:** 2016-02-01

**Authors:** Sen Wang, Farzam Javadpour, Qihong Feng

**Affiliations:** 1 Bureau of Economic Geology, Jackson School of Geosciences, The University of Texas at Austin, Austin, TX, United States; 2 School of Petroleum Engineering, China University of Petroleum (East China), Qingdao, China

## Abstract

We optimized potential parameters in a molecular dynamics model to reproduce the experimental contact angle of a macroscopic mercury droplet on graphite. With the tuned potential, we studied the effects of pore size, geometry, and temperature on the wetting of mercury droplets confined in organic-rich shale nanopores. The contact angle of mercury in a circular pore increases exponentially as pore size decreases. In conjunction with the curvature-dependent surface tension of liquid droplets predicted from a theoretical model, we proposed a technique to correct the common interpretation procedure of mercury intrusion capillary pressure (MICP) measurement for nanoporous material such as shale. Considering the variation of contact angle and surface tension with pore size improves the agreement between MICP and adsorption-derived pore size distribution, especially for pores having a radius smaller than 5 nm. The relative error produced in ignoring these effects could be as high as 44%—samples that contain smaller pores deviate more. We also explored the impacts of pore size and temperature on the surface tension and contact angle of water/vapor and oil/gas systems, by which the capillary pressure of water/oil/gas in shale can be obtained from MICP. This information is fundamental to understanding multiphase flow behavior in shale systems.

Shale—a typically fine-grained sedimentary rock having ultra-low permeability and previously regarded as inaccessible—has received extensive attention, owing to its enormous hydrocarbon reserves and economical production rate after fracking. The great success in North America has confirmed the potential of shale resources, leading to a worldwide ‘shale revolution’^1^,^2^. Recent experimental studies suggest that the pore network in a shale matrix consists of both the void space associated with mineral crystals and the intraparticle pores located within organic matter, the size of which ranges from a few micrometers to the nanometer scale (~2 nm)^3^. Because materials at the nanoscale show different properties from those they exhibit at the macroscopic scale, e.g., the enhanced flow in a carbon nanotube (CNT)^4^ and the superhydrophobicity of a textured surface^5^, the shale pore structure must be accurately characterized, with the ultimate goal of understanding the thermodynamic states, phase behavior, and transport properties of water/hydrocarbons in shale gas systems.

Approaches commonly employed to determine the pore size distribution (PSD) of shales include low-pressure adsorption (LPA) using N_2_ and CO_2_, mercury intrusion capillary pressure (MICP) measurement, nuclear magnetic resonance (NMR), and scanning electron microscopy (SEM) image analysis^3^,^6^,^7^. Among these methods, MICP is deemed a standard and metric determination of PSD, particularly in industry, because it is one of the few techniques by which pore sizes ranging over 4 to 5 orders of magnitude (~0.002–100 μm) can be probed using a single method^7^,^8^. The externally imposed pressure, *p*_*c*_, required to inject the nonwetting mercury into the pores with a particular radius, *r*, is typically given by the Washburn equation^9^, *p*_*c*_ = −2*γ*cos*θ*/*r*, where *γ* is the liquid-vapor surface tension of mercury and *θ* is the contact angle. During the interpretation of MICP, *γ* and *θ* are always assumed to be constant, and a series of arbitrary, unverified values, e.g., 480 mN/m and 140°, are used^10^. Taking into account that the surface tension of a liquid droplet is strongly dependent on the curvature^11^ and the contact angle also varies with the pore size^12^, we hypothesized that the common procedure for MICP analysis will lead to a significant error for shale pore characterization. On the basis of an experiment using controlled pore glass with a constant radius, Kloubek^10^ reported that their combined effect is negligible. However, his experimental material, amorphous glass, is quite different from shale. Considering that the thermophysical properties of a material at the nanoscale are dominated by solid-liquid interactions, one may doubt their applicability to shale. Therefore, study of the variations of surface tension and contact angle of mercury with shale pore size is vital for its accurate characterization. Moreover, this technique may serve as an efficient tool to estimate the capillary pressure of water/oil/gas of shales under sedimentary conditions. This knowledge is essential to gain insight into the science of multiphase flow in nanoporous material.

We optimized the pairwise potential between mercury and carbon to reproduce the macroscopic contact angle of a mercury droplet on graphite through molecular dynamics (MD). This potential model enabled us to study the wetting of liquid mercury in organic-rich shale nanopores. We found that the contact angle varies with pore size, geometry, and temperature. From a theoretical model, we predicted the curvature-dependent surface tension of liquid mercury droplets and suggested that it becomes smaller as droplet size decreases. We corrected the common interpretation procedure of MICP to account for the variation of contact angle and surface tension with pore size and showed that this correction improves the agreement of PSD derived from MICP and adsorption data, especially for pores having a radius of <5 nm. Finally, we discussed the effects of pore size and temperature on the contact angle and surface tension of water/vapor and oil/gas systems. Through the J-function in petrophysics, our results can be used to predict the capillary pressure of water/oil/gas in shales from MICP. To the best of our knowledge, this work is the first study of the size-dependent contact angle and surface tension of shales. Our results, which highlight the need for the calibration of contact angle and surface tension at the nanoscale, have implications for the characterization of shale pore structure but more generally for multiphase flow modeling in nanoporous materials.

## Results

### Determination of Hg-C interaction potential

When MD is employed as a predictive tool, validated interaction parameters are essential to ensure accuracy. In order to cooperate with the temperature-dependent potential between Hg atoms^13^ (see Methods), we optimized the pairwise interaction between mercury and carbon atoms to reproduce the experimentally measured macroscopic contact angle of a mercury droplet on a smooth graphite surface at 300 K. We used a 12-6 Lennard-Jones potential to describe the interaction^14^. The diameter *σ*_Hg-C,_ 3.321 Å, was determined by applying Lorentz-Berthelot combining rules, *σ*_*ij*_ = (*σ*_*ii*_+*σ*_*jj*_)/2, to the pair of *σ*_C-C_ = 3.407 Å and *σ*_Hg-Hg_ = 3.234 Å^14^. However, the potential well depth, *ε*_Hg-C_, should be optimized.

Because of the three-phase contact line, the microscopic contact angle determined from MD, *θ*, is different from the equilibrium contact angle at the macroscopic scale, *θ*_∞_. Their relationship is given by the modified Young’s equation^15^:


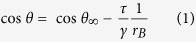


That is, cos*θ* is linearly dependent on the curvature of the contact line, 1/*r*_*B*_. Previous work shows that the line tension, *τ*, is on the order of 10^−12^ to 10^−10^ J/m and can be either positive or negative^16^,^17^. A positive *τ* indicates that the droplet base tends to contract, corresponding to a larger contact angle, whereas a negative value enhances the wettability^12^.

By varying the mercury droplet sizes in our MD simulations, we calculated the contact angle *θ* (see Methods) and determined the macroscopic value *θ*_∞_ through Eq. (1). Then *θ*_∞_ values were compared with the experimental contact angle, from which we adjusted *ε*_Hg-C_ to reproduce the macroscopic observation. We made three groups of simulations (see Supplementary Table S1, Group A1 to A3). In each group, we used the same potential parameters, but the number of mercury atoms of the droplets varied, ranging from 2,000 to 8,000. A potential set with *σ*_Hg-C_ = 3.321 Å and *ε*_Hg-C_/*k*_B_ = 14.7 K, which is the same as the electrowetting simulation performed by Chen *et al.*^14^, was used in Group A1. We kept *σ*_Hg-C_ unchanged and decreased *ε*_Hg-C_ by 30% (*ε*_Hg-C_/*k*_B_ = 10.29 K) in Group A2 but increased *ε*_Hg-C_ by 30% (*ε*_Hg-C_/*k*_B_ = 19.11 K) in Group A3.

**Figure 1:**
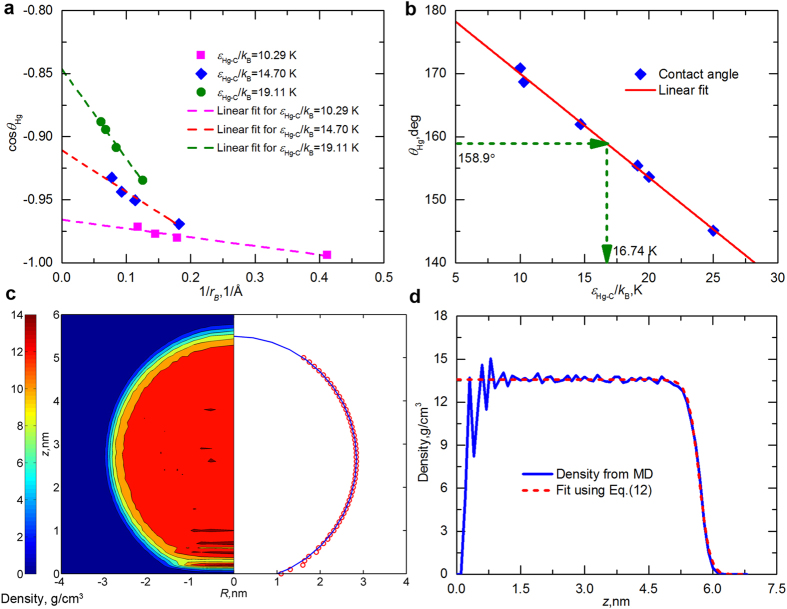
Determination of Hg-C interaction potential. (**a**) Cosine of the contact angle *θ* as a function of the reciprocal of the droplet base radius, 1/*r*_*B*_. The results were obtained from MD simulations using different mercury-carbon interaction parameters, i.e., *ε*_Hg-C_/*k*_B_ = 10.29, 14.70, and 19.11 K, for droplets consisting of an increasing number of mercury atoms (2,000 through 8,000). For each *ε*_Hg-C_, the intercept of the linear fit provides the macroscopic contact angle *θ*_∞_. (**b**) Contact angles of mercury droplets on graphite as a function of *ε*_Hg-C_. The solid line is a linear fit to the contact angles obtained from MD (4,000 mercury atoms). (**c**) Time-averaged density map (left) and the computation of contact angle by fitting a circle (solid line) to the points (open circles) of the equimolar dividing surface (right). In this case, *σ*_Hg-C_ = 3.321 Å and *ε*_Hg-C_/*k*_B_ = 16.74 K, resulting in a microscopic contact angle of 159°, which reproduces the macroscopic measurement (152.5°) of a mercury droplet on graphite at 300 K. (**d**) Mercury density profile (solid line) at the axis of symmetry (*R* = 0) as a function of *z*. The dashed line gives the fit with Eq. (12).

In Fig. 1a, we present the variation of the cosine of the microscopic contact angle, cos*θ*, with the curvature of the droplet base, 1/*r*_*B*_. The linear fit to the results of each group is also superimposed. From Eq. (1), we know that extrapolating these lines to the limit of 1/*r*_*B*_ → 0, i.e., their intercepts on the *y* axis, gives the macroscopic contact angles corresponding to different Hg-C potentials. The computed *θ*_∞_ values are 164.98°, 155.62°, and 147.82° for the weaker, normal, and stronger interaction, respectively. The deviations between *θ*_∞_ and *θ* for the droplets composed of 4,000 mercury atoms are −3.7°, −6.36°, and −7.57°. This great discrepancy suggests that the effect of line tension should not be ignored at the nanoscale. Therefore, in calibrating *ε*_Hg-C_, we considered the line tension and adjusted the target value from the macroscopic measurement, 152.5°^18^, to its counterpart of 158.9° at the microscopic scale. It is worth noting that although Chen *et al.*^14^ also recovered the experimental contact angle by varying *ε*_Hg-C_, they assumed 4,000 mercury atoms are sufficient to represent a macroscopic droplet and failed to take into account the contribution of line tension. Another drawback of their force field is that the potential model they used for mercury can only be applied at 300 K, because the temperature-dependent feature of Hg-Hg interatomic potential was not accounted for^13^. These issues were fully solved in our model.

Now we will determine the interaction parameters between mercury and carbon, *ε*_Hg-C_. In all of these simulations, we used 4,000 mercury atoms and increased *ε*_Hg-C_/*k*_B_ from 10 to 25 K (see Supplementary Table S1, Group A4 and Cases #2, #6, #10). As expected, the contact angle monotonically decreases with the increment of the pairwise interaction (Fig. 1b). Also included in Fig. 1b is a linear fit to all of the data points, from which we obtained *θ* =  −1.647*ε*_Hg-C_+186.473 (*R*^2^ = 0.98). Thus, a macroscopic contact angle of 152.5° requires an interaction parameter of *ε*_Hg-C_/*k*_*B*_ = 16.74 K to obtain the corresponding microscopic value of 158.9°. To validate our result, we performed a simulation using the parameters *σ*_Hg-C_ = 3.321 Å and *ε*_Hg-C_/*k*_B_ = 16.74 K. In good agreement with our prediction, the optimized potential led to a microscopic contact angle of 159.03° for a droplet consisting of 4,000 mercury atoms (Fig. 1c). We also found that the density remains almost constant in the central region of the droplet, whereas in the vicinity of the solid substrate, intense fluctuations are present. To show this result more clearly, we depicted the density profile along the symmetry axis (*R* = 0) of the droplet in Fig. 1d and also the fit result using the sigmoidal function (see Methods). The estimated density of liquid mercury is 13.563 g/cm^3^, consistent with the experimental value, 13.533 g/cm^3 ^19, at 300 K. Adjacent to the graphite surface, the strong oscillations on the density profile, which indicate a layering structure of mercury atoms, conform with results of previous work^20^. In subsequent simulations, we used the optimized potential to study the contact angle of mercury confined in shale nanopores.

### Contact angles of mercury in shale nanopores

The effects of pore size, geometry, and temperature on the contact angle of mercury inside shale nanopores were examined (see Methods). We considered mercury droplets confined in both circular and slit-shaped pores. Similar to the smooth graphite surface, we estimated the contact angle by fitting a circle to the liquid-vapor interface. The simulation details and results are summarized in Supplementary Table S2.

**Figure 2:**
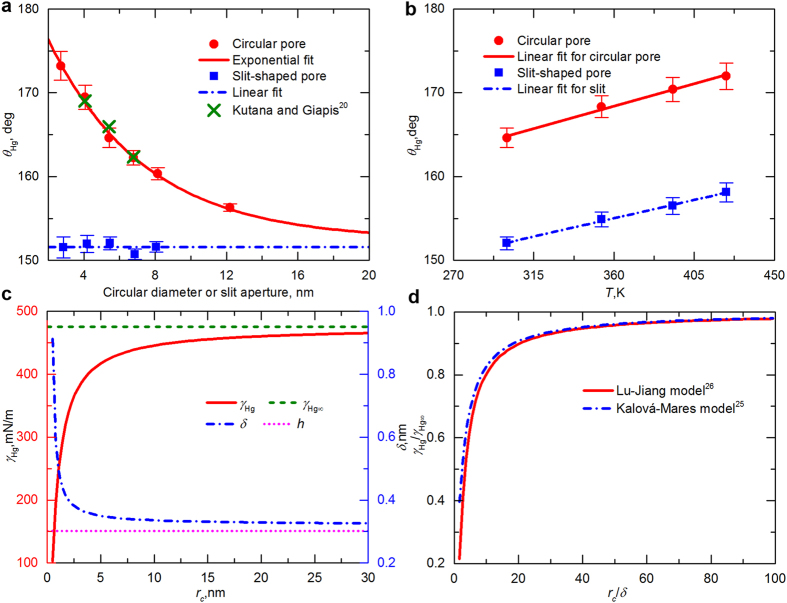
Contact angles of mercury in shale nanopores and its curvature-dependent surface tension. (**a**) Effects of pore size on the contact angle of mercury in circular and slit-shaped pores at *T* = 300 K. The green cross points indicate the results obtained by Kutana and Giapis^20^. (**b**) Effects of temperature on the contact angle of mercury in 5.4-nm circular and slit-shaped pores. (**c**) Variations of the surface tension, *γ*_Hg_, and the Tolman’s length, *δ*, of mercury with the droplet radius. The green dashed and magenta dotted lines represent the surface tension, *γ*_Hg∞_, of a planar interface (*r* = ∞) and the atomic diameter, *h*, respectively. (**d**) Surface tension, *γ*/*γ*_∞_, as a function of size, *r*_*c*_/*δ*, of mercury droplets. The coincidence of the Lu-Jiang and Kalová-Mareš models validates our results. Error bars show standard deviation computed from four replicates.

Figure 2a shows the computed contact angle of circular pores of different sizes. One can see that the contact angle increases as pore size decreases, indicating a more mercurophobic behavior and a positive line tension (Eq. (1)). This trend is consistent with the effect of drop size upon the contact angle of mercury on smooth graphite (Fig. 1a). Moreover, our results for pores having diameters of 4.07, 5.42, and 6.78 nm are close to those obtained by Kutana and Giapis^20^. To calibrate the mercury intrusion technique, we got an empirical function for *θ *~ *r* using exponential fitting:


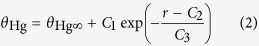


In this equation, *θ*_Hg∞_ = 152.446°, and the parameters *C*_1_–*C*_3_ are 18.345, 1.719, and 2.7117, respectively. Figure 2a shows that the dependence of contact angle on pore size for mercury confined in shale nanopores can be well characterized by Eq. (2) (*R*^2^ = 0.96). Corresponding to an infinite droplet, i.e., a vanishing wall curvature, the contact angle converges to a value of 152.446°, which agrees with the experimental result for a mercury droplet on a graphene sheet, 152.5° (Table 1).

The variation of contact angle with slit aperture is also included in Fig. 2a. In contrast with the contact angles in circular pores, the contact angles of mercury droplets confined in slits are independent of pore size, because under this condition the three-phase contact line tends to be straight, resulting in a negligible effect of line tension (Eq. (1)). Thus, within the range of uncertainty, the average value indicated by the dashed-dot line, 151.57°, is approximately equal to the macroscopic measurements.

The contact angles of mercury droplets in circular and slit-shaped shale nanopores increase linearly with temperature (Fig. 2b). Over the range explored, from 300 to 423 K, the contact angle increases by approximately 6°, and no significant effect of pore geometry is shown on the variation of contact angle with temperature. This conclusion is supported by the experiments of Ellison *et al.*^21^, who measured the contact angles of mercury on different materials (e.g., steel, glass, fused quartz, etc.) and suggested that the relationship of *θ* ~ *T* is positive for all the solid-mercury system, and the greatest increase happens for Teflon (~15° when increasing *T* from 300 to 423 K).

### Curvature-dependent surface tension of mercury

Study of the variation of surface tension with droplet size was pioneered by Gibbs^22^. He predicted that surface tension will decrease monotonically as the droplet curvature increases and suggested that this effect would be negligible for macroscopic droplets but become prominent at small radii. Tolman^11^ provided a rigorous analysis and established the well-known Gibbs-Tolman-Koenig-Buff (GTKB) equation (see Methods). The Tolman’s length, *δ*, which means the separation between the equimolar dividing surface, *R*_*e*_, and the surface of tension, *R*_*s*_, i.e., *δ* = *R*_*e*_ − *R*_*s*_, was defined in this equation. If *δ* is known, the surface tension can be estimated for a given droplet. However, the Tolman’s length is not expected to be a trivial function of droplet radius^23^. Up to now, there is even widespread controversy about the sign of *δ*^24^. Other models built by simplifying the GTKB equation share the same limitation^25^. Therefore, a theoretical model proposed by Lu and Jiang^26^, which is free of any adjustable parameter, was used to predict the dependence of surface tension on droplet radius *r*_*c*_:


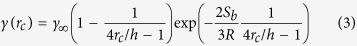


where *S*_*b*_ = *E*_0_/*T*_*b*_, and *E*_0_ is the enthalpy of vaporization, kJ/mol; *T*_*b*_ is the boiling point, K; *R* is the ideal gas constant, i.e., 8.314 J/(K·mol); and *h* is the effective atomic or molecular diameter, nm. All the parameters in the Lu-Jiang model are readily obtained from the thermodynamic properties of a given element^27^. A comparison of the results of this model with the simulation results shows that it provides a reasonably good prediction (see Supplementary Information).

Using the Lu-Jiang model, we estimated the surface tension of mercury droplets having various radii (Fig. 2c). The corresponding parameters are listed in Supplementary Table S3. With the increment of droplet size, the surface tension increases monotonically and converges to 475.5 mN/m for a planar surface. In particular, the sharp change mainly appears at *r*_*c*_ < 10 nm. When we increase *r*_*c*_ from 20 to 30 nm, the surface tension only increases 1.06%. However, an increase in droplet radius from 1 to 10 nm leads to a 76.8% rise in mercury surface tension. Because a large proportion of pores in a shale matrix are in this range (1–10 nm), interpreting the mercury intrusion data by ignoring the variation of mercury surface tension with droplet size will result in a large error. In Fig. 2c, the Tolman’s length computed through the Lu-Jiang model (see Methods) is also shown as a function of the droplet radius of mercury. As suggested by Tolman^11^, *δ* decreases with *r*_*c*_ and tends to approach the atomic diameter of mercury, *h*, at the infinite limit of droplet radius (*r*_*c*_ → ∞). The dimensionless relationship between mercury surface tension, *γ*_Hg_/*γ*_Hg∞_, and the droplet size, *r*_*c*_/*δ*, is depicted in Fig. 2d. For comparison, the result predicted using the analytical solution of the GTKB equation with minimum assumptions, that is, the Kalová-Mareš model^25^ (see Methods), is also included. Owing to the unknown *δ*, this model cannot be directly applied to calculate the surface tension of liquid droplets. However, the function between *γ*/*γ*_∞_ and *r*_*c*_/*δ* can be estimated from this model. The coincidence of the Lu-Jiang model (Eq. (3)) and the Kalová-Mareš model validates our prediction of the size-dependent surface tension of mercury droplets.

### Calibration of mercury intrusion technique

We have demonstrated that the contact angle, *θ*_Hg_, and liquid-vapor surface tension, *γ*_Hg_, of mercury droplets strongly depend on the pore size. Therefore, the conventional interpretation technique for MICP, in which *θ*_Hg_ and *γ*_Hg_ are generally assumed to be constant, should be corrected to take into account both the variation of contact angle with pore radius (Eq. (2)) and the change of surface tension as a function of the droplet curvature (Eq. (3)). Note that the meaning of *r*_*c*_ in Eq. (3) is different from that of *r* in Eq. (2): *r*_*c*_ represents the curvature radius of the droplet surface, whereas *r* stands for pore radius. Their relationship is described by^10^


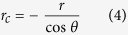


Combining Eqs. (2)–(4), 3, 4 into the Washburn equation^9^, which is commonly employed to correlate the mercury intrusion capillary pressure to the pore radius, we obtained a nonlinear formulation:


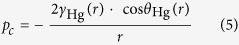


In comparison with the original equation, *γ*_Hg_ and *θ*_Hg_ are functions of *r* instead of being constant. For a given intrusion pressure, the pore radius can be determined from Eq. (5) via the Newton-Raphson iteration. If *f*(*r*) is defined as *f*(*r*) = *p*_*c*_*r*+2*γ*_Hg_(*r*)cos*θ*_Hg_(*r*), the solution of *f*(*r*) = 0 gives the pore radius corresponding to *p*_*c*_. The derivative of *f*(*r*) is


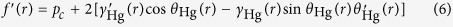



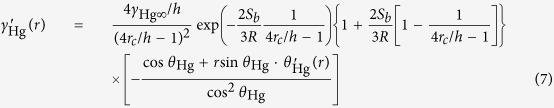



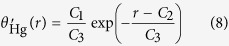


With an initial choice of *r* (2.0 nm is used here), the algorithm can be iteratively applied until a sufficiently accurate value is reached,


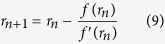


**Figure 3:**
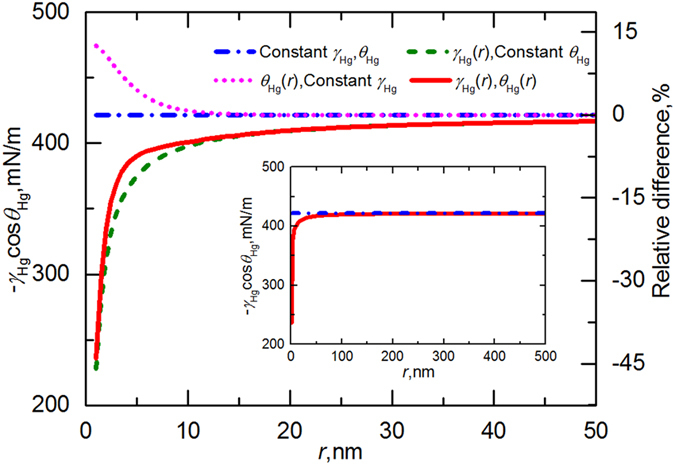
Value of −*γ*_Hg_cos*θ*_Hg_ calculated from different models as a function of pore radius *r*. *θ*_Hg_(*r*) and *γ*_Hg_(*r*) represent the dependence of contact angle and surface tension on pore size taken into account in the model. Constant *θ*_Hg_ and *γ*_Hg_ indicate that the parameters remain unchanged, i.e., *θ*_Hg_ = 152.5°, and *γ*_Hg_ = 475.5 mN/m. To separate the contribution of *θ*_Hg_ and *γ*_Hg_, the results computed by altering only one parameter are also shown. The inset gives −*γ*_Hg_cos*θ*_Hg_ versus *r* in the range up to 500 nm. The right axis shows the relative difference defined as (*G*′−*G*)/*G*, where *G*′ is the value of −*γ*_Hg_cos*θ*_Hg_ calculated from each model, and *G* corresponds to that obtained from the original Washburn equation.

To explore the effect of pore size on the analysis of MICP, we calculated the variation of −*γ*_Hg_cos*θ*_Hg_ against the pore radius *r* using the original Washburn equation and Eq. (5). The results are presented in Fig. 3, together with the independent contribution of *θ*_Hg_ and *γ*_Hg_ obtained by altering only one parameter while keeping the other one unchanged. The contact angle and surface tension show reverse effects on the value of −*γ*_Hg_cos*θ*_Hg_. For a decreased *r*, the contact angle gradually increases, causing a larger estimation of −cos*θ*_Hg_, whereas *γ*_Hg_ exhibits a downward trend with decreasing pore size (Fig. 3). In the model considering both effects, the monotonically incremental tendency of −*γ*_Hg_cos*θ*_Hg_ upon the pore radius reveals that the variation is mainly controlled by the surface tension and the influence of cos*θ*_Hg_ is limited. In the inset, we show −*γ*_Hg_cos*θ*_Hg_ as a function of *r* in the range up to 500 nm. For the pores having radii greater than 100 nm, the values of −*γ*_Hg_cos*θ*_Hg_ obtained from Eq. (5) and the original equation almost coincide with each other, suggesting that the impact of pore size on −*γ*_Hg_cos*θ*_Hg_ is negligible in conventional reservoirs (pore radius: 1–50 μm). This finding justifies the validation of using −*γ*_Hg_cos*θ*_Hg_ as a constant in the pore characterization of conventional sedimentary rocks. However, as indicated in Fig. 3, if the pore radius is smaller than 50 nm—the most common size in a shale matrix—there is a large deviation of the values of −*γ*_Hg_cos*θ*_Hg_ computed through the original Washburn equation and the value estimated by Eq. (5). We also compared the relative differences among these models (Fig. 3, right axis). The Washburn equation in its standard form is not valid for smaller pores and will yield a relative error of 8 to 44% when *r* < 5 nm. Previous studies confirm that at this scale, a material’s property may be completely distinct from the bulk value^4^,^5^,^28^,^29^; hence, precise interpretation of the PSD is important to evaluate the storage states and transport properties of oil/gas/water in shale pore networks, necessitating the calibration of traditional MICP analysis.

**Figure 4:**
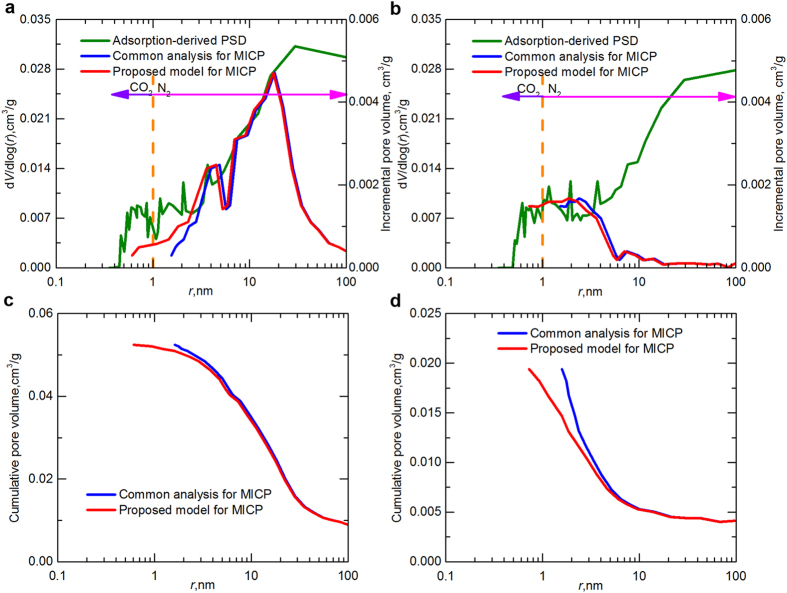
Correction of mercury intrusion technique. (**a**,**b**) Comparison of the PSD estimated from common interpretation of MICP, our proposed model for MICP, and the combined CO_2_/N_2_ adsorption-derived data. The samples were taken from the (**a**) Milk River Formation (Late Cretaceous) and (**b**) Barnett Shale gas play (Mississippian). The adsorption-derived PSD is scaled at the left, and the MICP data are scaled at the right. Results of the common analysis of MICP and the adsorption data are from Clarkson *et al.*^6^. The purple and magenta arrows indicate the range covered by CO_2_ and N_2_ adsorption, respectively. (**c**,**d**) Cumulative pore volume as a function of pore radius for the samples taken from the (**c**) Milk River Formation and (**d**) Barnett Shale.

Using high-pressure MICP, gas (N_2_ and CO_2_) adsorption, and small-angle/ultra-small-angle neutron scattering (SANS/USANS) techniques, Clarkson *et al.*^6^ measured the PSD of several shale samples taken from currently active plays in North America and compared the capability of these approaches to characterize the complex topology of shales. They reported that the PSD derived from MICP is inconsistent with the gas adsorption data (Fig. 4a,b), and the possible reason may be attributed to the compression of mineral particles at high pressure and the fact that MICP provides information on throats rather than the pore bodies. However, here we show that if the common analysis method for MICP is corrected to account for the variations of both the surface tension and the contact angle of mercury with the shale pore size, the agreement between MICP- and adsorption-derived PSD improves considerably, especially for pores having smaller radii (Fig. 4a,b). Comparisons of the cumulative pore volume (Fig. 4c,d) also reveal that the key feature that distinguishes the results of common analysis from results of our proposed model lies in pores having radii <10 nm. Our method not only improves the accuracy of determining shale PSD from mercury intrusion but also extends the potential suitability of MICP to characterize other nanoporous materials.

## Discussion

**Figure 5:**
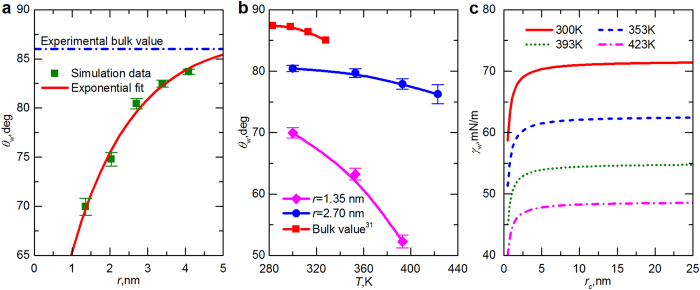
Contact angle and surface tension of water droplets in shale nanopores. (**a**) Effects of pore size on the contact angle of water in shale nanopores at *T* = 300 K. The dashed-dot line indicates the experimental contact angle of a macroscopic water droplet on a smooth graphite surface, i.e., 86° at 300 K. (**b**) Temperature variation of water contact angle in shale nanopores. The bulk values are taken from the study of Taherian *et al.*^31^. (**c**) Variation of water surface tension with droplet radius at different temperatures. Error bars show standard deviation computed from four replicates.

The variations of contact angle with pore size and temperature are also studied for water in shale nanopores through MD (see Methods). In contrast to the shale-mercury system, the contact angle of water decreases as the pore size becomes smaller (Fig. 5a); that is, the inner surface tends to be more hydrophilic. This discrepancy may be correlated to the different hydrophobicity of water and mercury on the same surface. The *θ* ~ *r* dependence for a shale-water system follows the same trend as the results reported for water in CNT by Werder *et al.*^30^. However, our computed contact angles are smaller than those in their evaluations because the water-carbon interaction parameters used by them (*σ*_O-C_ = 3.19 Å, *ε*_O-C_/*k*_B_ = 37.724 K) cannot reproduce the experimental measurement. This issue was further discussed by Werder *et al.*^12^, and a macroscopic value of 103.7° was obtained for this parameter set. We also found that the relationship between contact angle and pore size can be reasonably described by an exponential function (Fig. 5a). Therefore, we extrapolated our data exponentially to estimate the contact angle at infinite radius. The prediction, 87.33°, is in good agreement with the contact angle measured for a water droplet on graphite, i.e., 85.6° at 300 K^12^ (Table 1). Figure 5b shows the effect of temperature on the contact angle of water droplets in shale nanopores of various sizes. The variation of water contact angle on graphite with temperature, which was reported by Taherian *et al.*^31^, is also included. Note that their result for the contact angle at 298 K, 87.2°, is almost identical to our computation, i.e., 87.33°, at 300K. With increasing temperature, the contact angle exhibits a downward trend, indicating a more hydrophilic shale surface. In addition, the impact of pore size on the contact angle is more pronounced at higher temperatures: a decrease of 10.49° in the contact angle is caused by reducing the pore radius from 2.7 to 1.35 nm at 300 K, whereas the variation is 25.64° at 393 K. This conclusion is similar to the temperature effect upon the size-dependent contact angle of water droplets on a boron-nitride (BN) substrate^32^.

The Lu-Jiang model was employed to compute the liquid-vapor surface tension of water against the droplet size at various temperatures (Fig. 5c), because a comparison with the simulation data indicates that this model is more accurate (see Supplementary Information). In this model, the surface tension of bulk water versus temperature was adopted from Vargaftik *et al.*^33^. We note that for water, *h* in Eq. (3) represents the bond length between the oxygen atom and hydrogen atom^26^. At each temperature, the surface tension gradually increases with the droplet radius and starts to converge when *r* >5 nm. Owing to the lower surface tension of bulk water at a higher temperature, a smaller *γ*_w_ is exhibited for the same droplet when heated.

**Figure 6:**
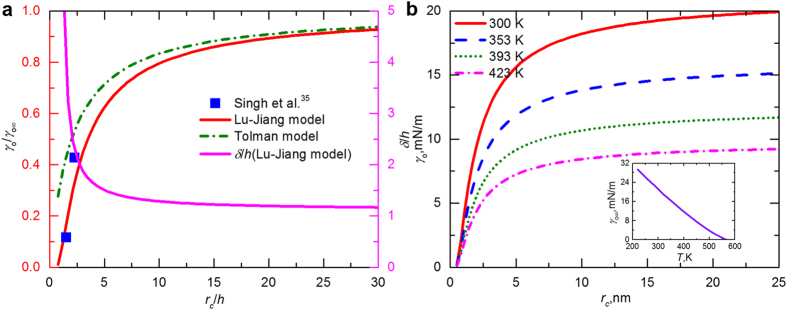
Curvature-dependent surface tension of *n*-octane. (**a**) Dependence of *γ*/*γ*_∞_ on *r*_*c*_/*h* for *n*-C_8_H_18_ estimated by the theoretical models and simulations by Singh *et al.*^35^. The solid and dashed-dot lines are predicted using the Lu-Jiang model (Eq. (3)) and the Tolman model (Eq. (14)), respectively. The relationship between *δ*/*h* and *r*_*c*_/*h* is also included; *γ*_o_/*γ*_o∞_ is read off the left axis, and *δ*/*h* off the right axis. (**b**) Variations of surface tension for *n*-C_8_H_18_ with droplet size at different temperatures. The inset shows the liquid-vapor surface tension of bulk octane as a function of temperature.

We further explored the dependence of oil surface tension on the droplet curvature in shale nanopores. Although the composition of petroleum is very complex, single-component *n*-octane is employed to represent the mixture because in our previous work^34^ we showed that the properties of *n*-C_8_H_18_ are very similar to those of oil produced from liquid-rich shales. To examine the applicability of the Lu-Jiang model to chain molecules, we calculated the dimensionless surface tension (*γ*/*γ*_∞_) of *n*-C_8_H_18_ as a function of the droplet radius (*r*_*c*_/*h*) using both the Tolman model (see Methods) and the Lu-Jiang model and then compared the results with the simulation data of Singh *et al.*^35^. By means of Grand Canonical Monte Carlo (GCMC) simulation, they studied the surface tension of alkanes confined in graphite pores and reported that the surface tension decreases manyfold compared with the bulk value. The parameters required for the theoretical models are listed in Supplementary Table S3. Even though Singh *et al.* only provided the surface tension of *n*-C_8_H_18_ at 2 and 3 nm, the Lu-Jiang model shows a better agreement with the simulations (Fig. 6a). Moreover, the values of *δ*/*h* estimated from the Lu-Jiang model decrease with droplet size and asymptotically converge to 1 in conformity with Tolman’s prediction, which validates the effectiveness of this model.

In order to determine the variation of surface tension, *γ*_o_, for *n*-C_8_H_18_ with the droplet radius, *r*_*c*_, the bulk value corresponding to a planar interface, *γ*_o∞_, must be known a priori. Through a differential capillary-rise method, Grigoryev *et al.*^36^ measured the surface tension of bulk *n*-C_5_H_12_, *n*-C_6_H_14_, *n*-C_7_H_16_, and *n*-C_8_H_18_ at temperatures ranging from the triple point to the critical point and proposed the following equation within the mean absolute error (MAE) of ±0.3 mN/m:


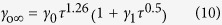


where *τ* = 1 − *T*/*T*_c_, and *T*_*c*_ is the critical temperature. For *n*-C_8_H_18_, *T*_*c*_, *γ*_0_, and *γ*_1_ are 568.82 K, 54.77 mN/m, and −0.0114, respectively. The inset in Fig. 6b shows the surface tension of bulk octane decreases at higher temperature. Therefore, for octane having the same droplet size, a dramatic decrease in the surface tension is caused by raising the temperature from 300 K to sedimentary conditions (Fig. 6b). If *T* remains constant, as the droplet size increases, the surface tension increases sharply when *r*_*c*_ < 10 nm, and then it tends to approach a constant value for bulk fluid.

In petrophysics, the J-function^37^, which combines the influences of porosity (*ϕ*), permeability (*k*), and contact angle (*θ*) into a dimensionless parameter for correlation, is commonly applied to describe rock heterogeneity. For a specific formation, the capillary pressure of different water/oil/gas systems can be reduced to a single curve of the J-function versus the liquid saturation. Thus, the measured data of MICP are normalized by the J-function to predict the capillary pressure of oil/gas or water/vapor^37^:


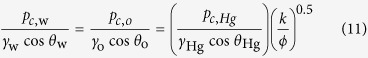


where the subscripts *w* and *o* stand for water and oil, respectively; the contact angle of an oil droplet on graphite is equal to zero. As mentioned above, the surface tension and contact angle are always assumed to be constant for conventional reservoirs. However, in the present work, we show that the surface tension and contact angle of mercury, water, and oil confined in shales greatly depend on the pore size and temperature. On the basis of our results, the capillary pressure curves for the other systems in shale can be accurately estimated from MICP by using Eq. (11). As the cornerstone of the mathematical models describing the multiphase flow and phase behavior, this information is crucial for reservoir numerical simulation, hydraulic fracturing design, and enhanced oil recovery in shale. In addition, the simulation technique that we used can be readily extended to other porous materials^30^. More generally, this study provided a framework for optimizing fluid-solid interaction potentials and exploring the variation of contact angle and surface tension with pore size, which may shed light on the modeling of multiphase flow in nanoporous media, i.e., translocation of water and proteins through biological nanopores^38^, sequestration of carbon dioxide and nuclear waste in geologic repositories^39^, gas separation and liquid purification with highly selective membranes^40^, and etc.

## Methods

### Molecular dynamics simulation

All the MD simulations were conducted by using Large-scale Atomic/Molecular Massively Parallel Simulator (LAMMPS)^41^. We adopted the force field proposed by Bomont and Bretonnet^13^ to describe the state-dependent pairwise interaction between mercury atoms. This model was adjusted to reproduce the experimental liquid densities along the liquid-vapor coexistence curve, and it has been successfully employed to recover the X-ray reflectivity experiments and predict the thermodynamic properties. For a mercury droplet on a graphite surface, the substrate consists of three prefect graphene sheets having an interlayer spacing of 3.35 Å and lateral dimensions of 103.3 × 102.3 Å^2^. Additional graphene layers are omitted because they are beyond the cutoff radius of 9 Å. Periodic boundary conditions are applied parallel to the basal surface of the substrate. All the carbon atoms are maintained fixed because the structure of the graphite framework is not expected to be significantly affected by mercury^20^. If the evaporated mercury atoms begin to move through the cell top, these atoms will be reflected toward the fluid region^20^. The fluid atoms are coupled to a Nosé–Hoover thermostat to maintain a constant temperature. We equilibrated the systems for 3.0 ns in an *NVT* ensemble (integration time step: 1 fs) and sampled the trajectories of the last 1.0 ns every 1 ps for analysis, from which the contact angle could be estimated. An overview of the simulation cases and results are summarized in Supplementary Table S1.

Once we determined the potential model between mercury and carbon, we examined the effects of pore size and temperature on the wetting of mercury droplets inside shale nanopores having different geometry: circular or slit-shaped (see details in Supplementary Table S2). We used three perfect graphene planes to represent the upper and lower surfaces of the organic slit in a shale matrix, and the circular pores are modeled ideally as a single-walled (*n, n*) carbon nanotube (SWCNT). Similar models have commonly been applied to study the adsorption and transport behavior of hydrocarbons in shale nanopores^34^,^42^. With the objective of encapsulating all the evaporated mercury atoms within the pores, we used periodic boundary conditions along the symmetry axis of the nanotube. Each case is equilibrated for 1.5 ns, followed by 2.0 ns for the production run (time step: 1 fs). All the other model implementations are equivalent to those of our simulations for mercury on the graphite surface.

To study the variations of contact angle with pore size and temperature for water droplets in shale nanopores, we performed MD simulations for SWCNT-H_2_O systems in which the pore diameters range from 2.7 to 8.1 nm. The interaction between water molecules is described by the extended Simple Point Charge potential (SPC/E). The O-H bond distance (1 Å) and the H-O-H angle (109.47°) are fixed with the SHAKE algorithm. The particle-particle particle-mesh (PPPM) solver is employed to calculate the long-range electrostatic interactions. For the water-carbon interaction, we used a Lennard-Jones 12-6 potential between the oxygen and the carbon atoms (*σ*_O-C_ = 3.19 Å, *ε*_O-C_/*k*_B_  = 47.17 K) within a cutoff distance of 10 Å. This parameter set was optimized by Werder *et al.*^12^ to recover the macroscopic contact angle (86°) of a water droplet on graphite at 300 K. We conducted 3.0-ns simulations in the *NVT* ensemble at 300, 353, 393, and 423 K through a Nosé–Hoover thermostat. Following an equilibration of 2.0 ns, the trajectories are collected during the remaining 1.0 ns.

### Determination of contact angle

We calculated microscopic contact angles from MD simulation trajectories using a circular fit to the liquid-vapor interface^12^. The time-averaged density distribution is first computed by means of a concentric cylindrical binning technique. For a mercury droplet on a graphite surface, we partitioned the droplet into several horizontal slabs at every 1-Å interval along the *z* axis. For each slab, the radial direction is discretized into *N*_bin_ cylindrical bins having the same base area of *δA* = 90 Å^2^; that is, the boundary for each bin is located at *R*_i_^2^ = *iδA*/*π* (*i* = 1, 2, …, *N*_bin_). We verified the reliability of this choice by varying both the height and base area of these bins, which led to an error within 1.0° for the computed contact angle. The average density profile in each horizontal slab, *ρ*(*R*), can be described by the sigmoidal function^12^


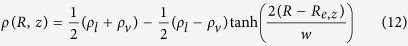


Here, *ρ*_*l*_ and *ρ*_*v*_ are the bulk density of liquid and vapor mercury, respectively (unit: g/cm^3^); *R*_*e,z*_ is the position of the equimolar dividing plane corresponding to the slab located at *z*; *w* is the thickness of the interfacial area, Å. Using a nonlinear fit, we determined the parameters in Eq. (12). We repeated the fitting for all the slabs and obtained a set of *R*_*e,z*_ points, which define a smooth and stable liquid-vapor interface at the equimolar dividing surface. Assuming a spherical geometry, the contact angle can be computed from a circular fit through the points of *R*_*e,z*_, The data points in both the vicinity of the solid surface (*z* < 8 Å) and the cap region of the droplet, e.g., *z* > 50 Å for 4,000 atoms, are excluded from the fit in order to eliminate the interference from density variations in the solid-liquid interface and the poor statistics in the cap. We then estimated the contact angle by extrapolating the fit to the substrate surface. To determine the contact angle under confinement, we used similar procedures. The points for equimolar dividing surfaces located within 5 Å from the solid wall and 3 Å from the symmetry axis are neglected for fit^12^,^20^.

### Theoretical model for surface tension

The GTKB equation is given by^11^


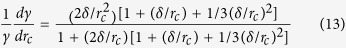


where *γ* is the surface tension of the liquid droplet, mN/m; *r*_*c*_ is the droplet radius, nm; and *δ* is the Tolman’s length, nm. For ease of application, Tolman simplified the GTKB equation by treating *δ* as a constant and ignoring the terms *δ*/*r*_*c*_ and *δ*^2^/*r*_*c*_^2^ in comparison with 1,


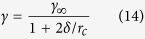


Further simplifications of the GTKB equation will bring other models^25^. Most recently, Kalová and Mareš^25^ evaluated the integral and got the dimensionless relationship between surface tension and droplet curvature,


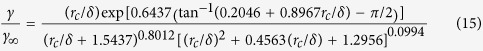


However, we cannot estimate the size-dependent surface tension of mercury droplets using these models directly, because the Tolman’s length, *δ*, is unknown. Tyson and Miller^43^ found that for a planar surface at the melting point, the ratio between the solid-vapor interfacial energy and the liquid-vapor surface energy is approximately a constant for metallic elements; that is, *γ*_sv∞_/*γ*_lv∞_ = *a* and *a* = 1.18±0.03. Then Lu and Jiang^25^ suggested that the derivation of the energy between solid and liquid is very small compared with that of solid and vapor or liquid and vapor. Therefore, they extended this function to the nanoscale, *γ*_sv_(*r*_*c*_)/*γ*_lv_(*r*_*c*_) = *a*. The relationship between the size-dependent solid-vapor interfacial energy and the bulk value can be described by^26^


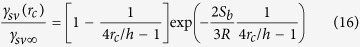


Lu and Jiang obtained


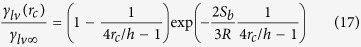


The asymptotic form of the Tolman’s length can be acquired by combing Eq. (17) and the Tolman model (Eq. (14))^26^:


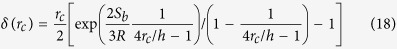


## Additional Information

**How to cite this article**: Wang, S. *et al.* Confinement Correction to Mercury Intrusion Capillary Pressure of Shale Nanopores. *Sci. Rep.* **6**, 20160; doi: 10.1038/srep20160 (2016).

## Supplementary Material

Supplementary Information

## Figures and Tables

**Table 1:** Comparisons between our MD simulation results and experimental contact angles for liquid droplets on smooth graphite at 300 K.

Liquid droplet	MD simulation	Experimental data	Ref.
Mercury	152.5 ± 0.5°	152.5 ± 2.0°	14,18
Water	87.3 ± 0.5°	85.6 ± 0.3°	12,44,45

The results are presented as mean ± standard deviation.
