# Supplementary material for: Confinement Correction to Mercury Intrusion Capillary Pressure of Shale Nanopores
Source: Sci Rep. 2016 Feb 1;6:20160. doi: 10.1038/srep20160 (PMC4735595; doi:10.1038/srep20160)
Supplement: Supplementary Information [file srep20160-s1.pdf]

Supplementary Information

# Confinement Corrections to Mercury Intrusion Capillary Pressure of Shale Nanopores

Sen Wang[1,2], Farzam Javadpour[1,*] & Qihong Feng[2]

[1]Bureau of Economic Geology, Jackson School of Geosciences, The University of Texas at Austin, Austin, TX, United States

[2]School of Petroleum Engineering, China University of Petroleum (East China), Qingdao, China

*Correspondence and requests for materials should be addressed to F.J. (email: farzam.javadpour@beg.utexas.edu)

This document contains the following sections:

Figure S1: Simulation snapshots of a mercury droplet (4,000 atoms) placed on a graphite surface ($\varepsilon_{Hg\text{-}C}/k_B$=20 K)

Figure S2: Simulation snapshots of a mercury droplet (2,520 atoms) confined in a circular pore having a diameter of 4.07 nm

Figure S3: Simulation snapshots of a mercury droplet (3,120 atoms) confined in a slit having an aperture of 4.19 nm

Table S1: Overview of the MD simulation cases for mercury droplets on a graphite surface

Table S2: Overview of the MD simulation cases for mercury droplets in shale nanopores

Text S1: Validation of the Lu-Jiang model

Table S3: Parameters used in the Lu-Jiang model

Figure S4: Dependence of $\gamma_{lv}/\gamma_{lv\infty}$ on $r_c/h$ estimated by simulations and theoretical models

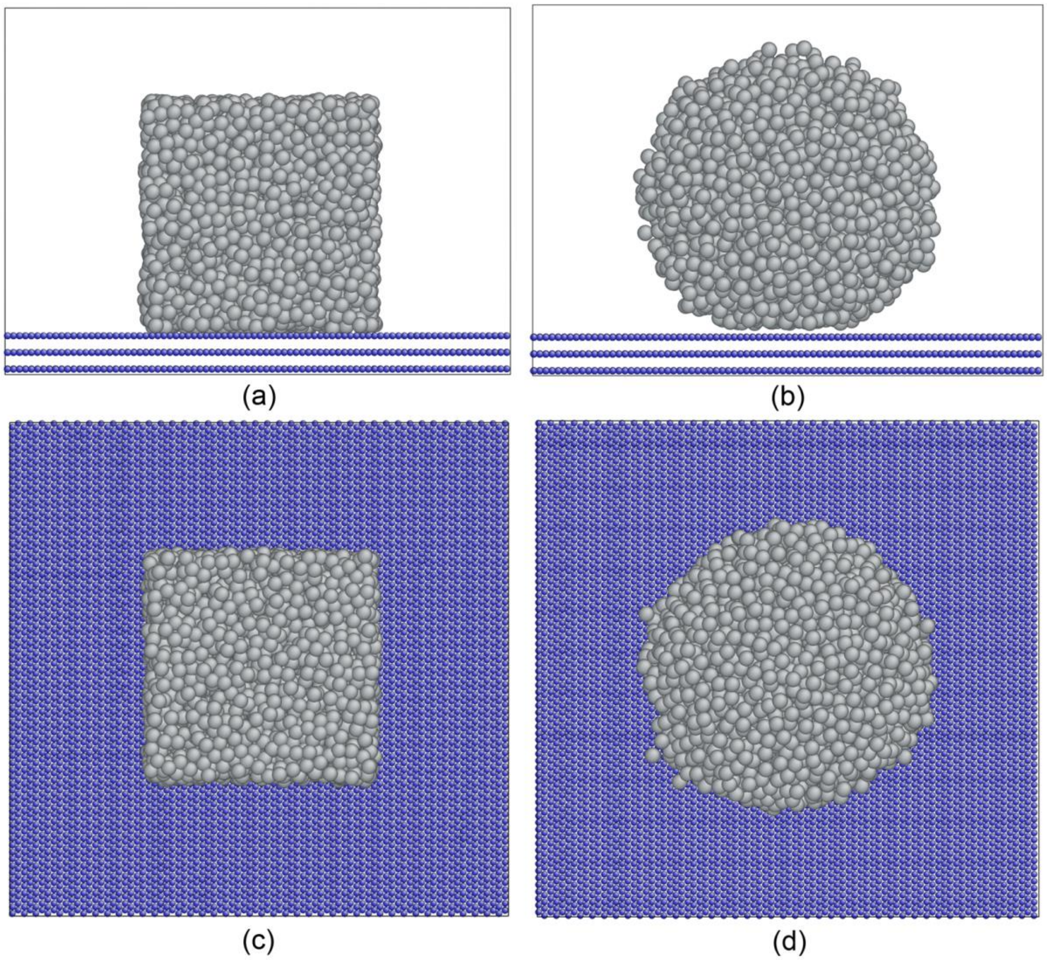


Figure S1. Simulation snapshots of a mercury droplet (4,000 atoms) placed on a graphite surface ($\varepsilon_{Hg\text{-}C}/k_B$=20 K): front (a and b) and top view (c and d) of the initial (left panels) and equilibrated (right panels) configuration. Color code: blue, carbon atom in graphene; silver, mercury.

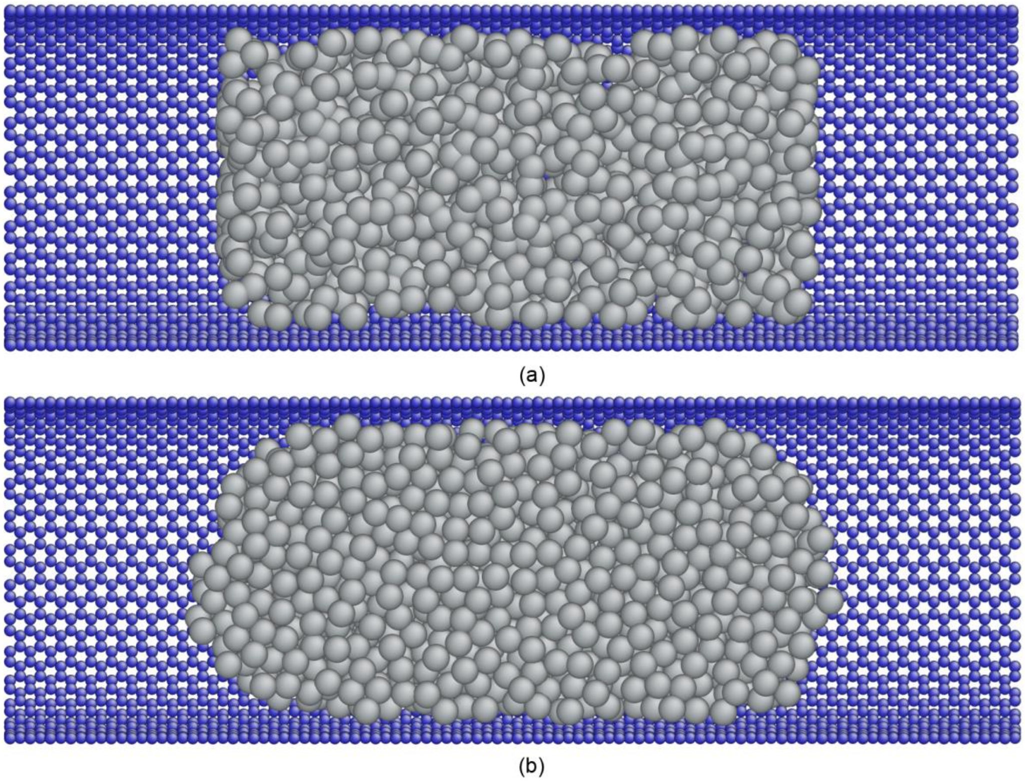


Figure S2. Simulation snapshots of a mercury droplet (2,520 atoms) confined in a circular pore having a diameter of 4.07 nm: the initial (a) and equilibrated (b) configuration. Parts of the carbon nanotube are not displayed for better visibility. Color code: blue, carbon atom; silver, mercury.

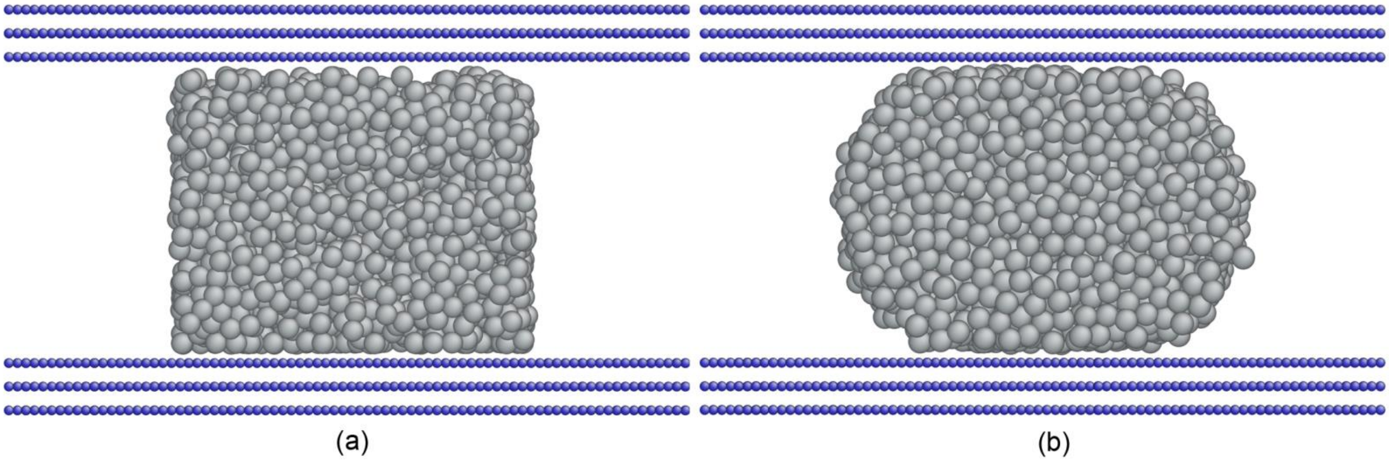


Figure S3. Simulation snapshots (front view) of a mercury droplet (3,120 atoms) confined in a slit having an aperture of 4.19 nm: the initial (a) and equilibrated (b) configuration. Color code: blue, carbon atom; silver, mercury.

Table S1. Overview of the MD simulation cases for mercury droplets on a graphite surface

| Group | Case # | $\sigma_{Hg\text{-}C}$, Å[1] | $\varepsilon_{Hg\text{-}C}/k_B$, K | $N_{Hg}$ | $r_B$, Å | $\theta$, deg |
|---|---|---|---|---|---|---|
| Group A1 | 1 | 3.321 | 14.7 | 2,000 | 5.50 | 165.85 |
| | 2 | 3.321 | 14.7 | 4,000 | 8.79 | 161.98 |
| | 3 | 3.321 | 14.7 | 6,000 | 10.75 | 160.77 |
| | 4 | 3.321 | 14.7 | 8,000 | 12.93 | 158.92 |
| Group A2 | 5 | 3.321 | 10.29 | 2,000 | 2.43 | 173.79 |
| | 6 | 3.321 | 10.29 | 4,000 | 5.57 | 168.68 |
| | 7 | 3.321 | 10.29 | 6,000 | 6.89 | 167.79 |
| | 8 | 3.321 | 10.29 | 8,000 | 8.46 | 166.37 |
| Group A3 | 9 | 3.321 | 19.11 | 2,000 | 7.95 | 159.26 |
| | 10 | 3.321 | 19.11 | 4,000 | 11.86 | 155.39 |
| | 11 | 3.321 | 19.11 | 6,000 | 14.60 | 153.51 |
| | 12 | 3.321 | 19.11 | 8,000 | 16.48 | 152.73 |
| Group A4 | 13 | 3.321 | 10 | 4,000 | 4.51 | 170.84 |
| | 14 | 3.321 | 20 | 4,000 | 12.68 | 153.62 |
| | 15 | 3.321 | 25 | 4,000 | 16.34 | 145.10 |
| Validation | 16 | 3.321 | 16.74 | 4,000 | 10.17 | 159.03 |

Table S2. Overview of the MD simulation cases for mercury droplets in shale nanopores

| Case # | Pore geometry | Pore size, nm | $T$, K | $N_{Hg}$ | $N_C$ | $\theta$, deg |
|---|---|---|---|---|---|---|
| C1 | Circular | 2.71 | 300 | 918 | 3,200 | 173.20 |
| C2 | Circular | 4.07 | 300 | 2,520 | 6,000 | 169.47 |
| C3 | Circular | 5.42 | 300 | 4,610 | 8,000 | 164.62 |
| C4 | Circular | 6.78 | 300 | 11,040 | 12,000 | 162.25 |
| C5 | Circular | 8.14 | 300 | 20,270 | 15,600 | 160.34 |
| C6 | Circular | 12.20 | 300 | 61,750 | 25,200 | 156.29 |
| C7 | Circular | 5.42 | 353 | 4,610 | 8,000 | 168.33 |
| C8 | Circular | 5.42 | 393 | 4,610 | 8,000 | 170.39 |
| C9 | Circular | 5.42 | 423 | 4610 | 8,000 | 171.97 |
| S1 | Slit | 2.86 | 300 | 1,670 | 7,200 | 151.53 |
| S2 | Slit | 4.19 | 300 | 3,120 | 9,600 | 151.96 |
| S3 | Slit | 5.48 | 300 | 5,770 | 10,800 | 152.04 |
| S4 | Slit | 6.86 | 300 | 7,750 | 12,000 | 150.73 |
| S5 | Slit | 8.07 | 300 | 11,625 | 14,400 | 151.61 |
| S6 | Slit | 5.48 | 353 | 5,770 | 10,800 | 154.87 |
| S7 | Slit | 5.48 | 393 | 5,770 | 10,800 | 156.49 |
| S8 | Slit | 5.48 | 423 | 5,770 | 10,800 | 158.11 |

**Text S1: Validation of the Lu-Jiang model**

In order to validate the Lu-Jiang model[2], the comparisons with the simulation results of Samsonov et al.[3,4] for three different materials are shown in Fig. S4. Also included in this figure are the predictions from the Tolman model with the assumption $\delta$=$h$. We summarized the input parameters in Table S3. Figure S4 indicates that the surface tension estimated from Eq. (17) is in good agreement with results of the simulation data. Both the Lu-Jiang model and the Tolman model show the surface tension decreases for smaller droplets, consistent with Gibbs's prediction. We also notice that the Lu-Jiang model is more accurate than the Tolman model.

Table S3. Parameters used in the Lu-Jiang model

| Materials | $h$, nm | $\gamma_{lv\infty}$, mN/m | $E_0$, kJ/mol | $T_b$, K | $S_b$=$E_0$/$T_b$, J/(mol K) |
|---|---|---|---|---|---|
| Al | 0.286[5] | 915[3] | 293[5] | 2,792[6] | 104.94 |
| Na | 0.372[5] | 208[3] | 97.7[5] | 1,156[6] | 84.52 |
| $H_2O$ | 0.096[7] | 71.67[8] | 13.6[8] | 373[7] | 36.46 |
| Hg | 0.302[5] | 475.5[9] | 59.2[5] | 629.88[5] | 93.99 |
| $n$-$C_8H_{18}$ | 0.655[10] | 8.385[11] | 39.4[12] | 398.7[12] | 98.82 |

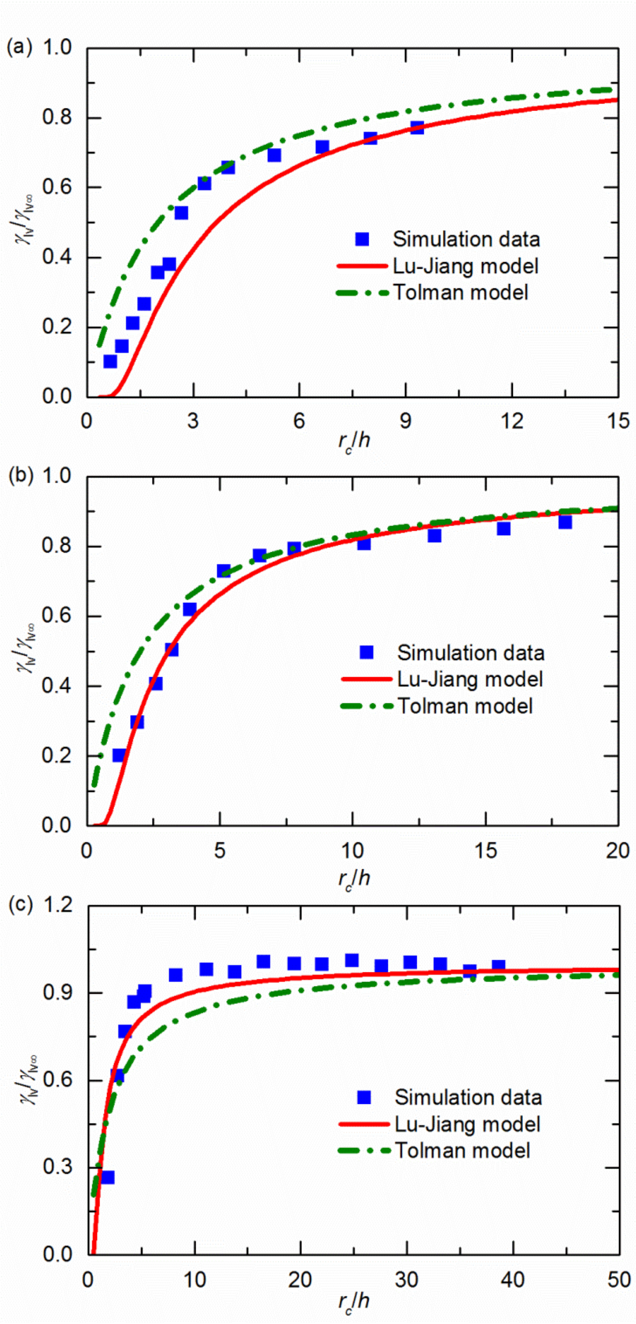


Figure S4. Dependence of $\gamma_{lv}/\gamma_{lv\infty}$ on $r_c/h$ estimated by simulations and theoretical models. (a) Aluminum (Al) droplets at the melting temperature; (b) Sodium (Na) droplets at the melting temperature; (c) Water ($H_2O$) droplets at room temperature. The simulation data (scatter points) are taken from work by Samsonov et al.[3,4]. The solid and dashed lines are predicted using Eqs. (17) and (14), respectively. The validation can also be found in the article by Lu and Jiang[2]. However, the simulation data of Na droplets are not correct in their figure[4].

## References


1. Kutana, A. & Giapis, K. P. Contact angles, ordering, and solidification of liquid mercury in carbon nanotube cavities. *Phys. Rev. E* **76,** 195444 (2007).
2. Lu, H. M. & Jiang, Q. Size-dependent surface tension and Tolman's length of droplets. *Langmuir* **21,** 779–781 (2005).3. Samsonov, V. M., Bazulev, A. N. & Sdobnyakov, N. Y. Rusanov's linear formula for the surface tension of small objects. *Dokl. Phys. Chem.* **389,** 83–85 (2003).
4. Samsonov, V. M., Shcherbakov, L. M., Novoselov, A. R. & Lebedev, A. V. Investigation of the microdrop surface tension and the linear tension of the wetting perimeter on the basis of similarity concepts and the thermodynamic perturbation theory. *Colloid Surface A* **160,** 117–121 (1999).
5. WebElements Periodic Table. Available at: http://www.webelements.com/
6. Tyson, W. R. & Miller, W. A. Surface free energies of solid metals: Estimation from liquid surface tension measurements. *Surf. Sci.* **62,** 267–276 (1977).
7. Dewar, M. J., Zoebisch, E. G., Healy, E. F. & Stewart, J. J. Development and use of quantum mechanical molecular models. 76. AM1: a new general purpose quantum mechanical molecular model. *J. Am. Chem. Soc.* **107,** 3902–3909 (1985).
8. Dean, J. A. *Lange's Handbook of Chemistry* (New York: McGraw-Hill, 1985).
9. Kozin, L. F. & Hansen, S. C. *Mercury Handbook: Chemistry, Applications and Environmental Impact* (Cambridge: Royal Society of Chemistry, 2013).
10. Ben-Amotz, D. & Herschbach, D. R. Estimation of effective diameters for molecular fluids. *J. Phys. Chem.* **94,** 1038–1047 (1990).
11. Grigoryev, B. A., Nemzer, B. V., Kurumov, D. S. & Sengers, J. V. Surface tension of normal pentane, hexane, heptane, and octane. *Int. J. Thermophys.* **13,** 453–464 (1992).
12. National Institute of Standards and Technology. Thermophysical properties of fluid systems. 2011; (http://webbook.nist.gov/chemistry/fluid/).
